# Predictive Value of Routine Peripheral Blood Biomarkers in Alzheimer’s Disease

**DOI:** 10.3389/fnagi.2019.00332

**Published:** 2019-12-05

**Authors:** Xiaoyu Dong, Jianfei Nao, Jile Shi, Dongming Zheng

**Affiliations:** Department of Neurology, Shengjing Hospital of China Medical University, Shenyang, China

**Keywords:** Alzheimer disease, biomarkers, blood routine, mild cognitive impairment, aging

## Abstract

**Background:**

Biomarker screening is of major significance for the early diagnosis and prevention of Alzheimer’s disease (AD). Routine peripheral blood parameters are easy to collect and detect, making them ideal potential biomarkers. Thus, we aimed to evaluate the parameters from routine blood as potential biomarkers for AD.

**Methods:**

We enrolled 56 AD patients, 57 mild cognitive impairment (MCI) patients, and 59 healthy elderly controls. Receiver operating characteristic (ROC) curves were used to assess the diagnostic values of routine blood biomarkers in patients with cognitive impairment.

**Results:**

There were significant differences in eight parameters between the groups. Logistic regression revealed that the neutrophil% (odds ratio (OR) 1.34, 95% confidence interval [CI] 1.03–1.75, *p* = 0.031) and neutrophil-to-lymphocyte ratio (NLR; OR 6.27, 95% CI 3.98–9.82, *p* = 0.003) differentiated AD patients and controls (areas under the curve [AUCs], 0.728 and 0.721) and that the NLR (OR 1.93, 95% CI 1.07–3.47, *p* = 0.028) and mean platelet volume (OR 1.67, 95% CI 1.04–2.70, *p* = 0.036) differentiated MCI patients and controls (AUCs, 0.60 and 0.638). There were no effective diagnostic biomarkers to distinguish AD from MCI.

**Conclusion:**

Some routine blood biomarkers may correlate with cognitive impairment. Analysis of these biomarkers, such as the NLR, may be useful for the identification of patients with cognitive impairment.

## Introduction

Alzheimer’s disease (AD), characterized by a progressive decline in memory and cognitive ability, is the most common neurodegenerative disease and severely affects the daily living abilities of the elderly. With increased global aging, AD has become one of the most important medical and social issues in the world (Morgan et al., 2019). The prevalence of AD in China is currently about 5% (Zhou et al., 2019). According to the latest World Report on AD, nearly 1 million Alzheimer’s patients are expected to be added each year by the year 2050.

Decades before the onset of dementia symptoms, a series of pathological changes occur in patients. When the symptoms of dementia eventually appear, the AD pathology has already entered the advanced stage (Reiman et al., 2011). Drugs and other interventions at this stage are unable to achieve satisfactory results, and several clinical trials of newly developed drugs for AD have failed in recent years (Honig et al., 2018). Some researchers have proposed that the incidence of AD can be reduced and the development of the disease delayed by advancing the intervention time and by enabling early prevention and treatment at the “preclinical” stage before its onset (McDade and Bateman, 2017).

Early detection, diagnosis, and treatment are of major clinical significance for the prevention and treatment of AD. For the early diagnosis of AD, screening of suitable biomarkers is vital. Compared with cerebrospinal fluid (CSF), blood samples are easier to collect and more repeatable. Therefore, screening of potential early diagnostic markers from blood is of particular importance. β-amyloid (Aβ) and tau protein in peripheral blood are the most studied blood markers. McDade and Bateman (2017) found that patients with high expression of Aβ42 in peripheral blood had an increased risk of dementia after 5 years. Assini et al. (2004) showed that women with mild cognitive impairment (MCI) had elevated Aβ42 in peripheral blood and suggested that elevated levels of Aβ42 and a decreased Aβ42/Aβ40 ratio in peripheral blood were risk factors for AD. Recently, a large study led by Fiandaca et al. (2015) showed that the tau concentration in peripheral blood was higher in patients with AD and was significantly correlated with future cognitive decline. However, the relatively low levels of Aβ and tau proteins in peripheral blood necessitate more sensitive detection techniques and increase detection costs, which restrict their application as diagnostic markers of AD.

Previous studies have shown that many routine peripheral blood parameters may be novel inflammatory markers and may be associated with the onset or prognosis of central nervous system diseases. For example, Schuss et al. (2018) showed that white blood cell count at admission was associated with poor prognosis in patients with subarachnoid hemorrhage. Elevated neutrophil and lymphocyte counts are considered to be a risk factor for secondary brain injury in patients with cerebral hemorrhage and are associated with poor short-term prognosis (Nguyen et al., 2007; Li et al., 2019). The monocyte count is correlated with 30-day mortality in patients with cerebral hemorrhage (Adeoye et al., 2014). Pikija et al. (2018) suggested that a neutrophil-to-lymphocyte ratio (NLR) elevation was associated with hemorrhagic transformation in patients with acute cerebral infarction. Arikanoglu et al. (2013) found that mean platelet volume (MPV) was correlated with mortality in patients with acute cerebral infarction. Platelet distribution width (PDW) and MPV can independently predict 90-day outcomes in stroke patients receiving thrombolysis (Xie et al., 2019). Dagistan and Cosgun (2019) suggested that MPV may be a predictor of dementia in elderly patients.

Considering the role of inflammation in the pathogenesis of AD, we hypothesized that routine blood biomarkers in AD patients could have diagnostic and predictive value. If confirmed, these routine blood biomarkers could be informative plasma markers for the diagnosis, stratification, and prediction of disease progression and/or may be used as proof of a response of MCI and AD to intervention. For this study, we obtained the routine blood parameters of 56 AD and 57 MCI patients attending a cognitive impairment clinic. At the same time, 59 healthy elderly people matched by age and sex were selected as controls. We analyzed the biomarkers and further validated our hypothesis using these samples.

## Methods and Measures

### Study Population

The case group selected in this study comprised patients with AD and MCI who visited the neurology ward or cognitive impairment clinic of ShengJing Hospital affiliated to China Medical University from September 2016 to September 2018. Healthy controls matched with the AD and MCI patients were recruited in outpatient clinics. Inclusion criteria were as follows: age >60 years old, intact visual and auditory function, and ability to complete the memory scale test. AD was diagnosed according to American Society of Neurology, Language Disorder and Stroke-Alzheimer’s Disease and Related Diseases Working Group (NINCDS-ADRDA) criteria for the diagnosis of “probable Alzheimer’s disease” (McKhann et al., 2011). MCI was diagnosed in accordance with Petersen’s criteria (Petersen, 2004). In terms of exclusion criteria, because the purpose of the study was to analyze routine blood parameters, it was necessary to exclude diseases associated with acute or chronic infectious diseases, rheumatic immune diseases, malignant tumors, severe liver and kidney diseases, blood system diseases, and endocrine system diseases that may affect routine blood indicators, as well as other neurological disorders that can lead to cognitive impairment; patients with a family history of dementia were also excluded.

### Routine Blood Testing

Fasting blood samples were obtained via sterile venipuncture after participants had fasted overnight between 5:00 and 6:00 a.m. Blood samples were immediately sent to the hospital’s clinical laboratory for analysis. Routine blood parameters were analyzed using an automatic hematology analyzer (Coulter LH 750; Beckman Coulter Inc., United States). White blood cell, neutrophil, lymphocyte, monocyte, erythrocyte, and platelet counts were measured by standard laboratory methods, as well as the neutrophil%, lymphocyte%, and monocyte% and the mean corpuscular volume (MCV), mean corpuscular hemoglobin (MCH), red cell distribution width (RDW), MPV, and PDW. The NLR, lymphocyte-to-monocyte ratio (LMR), and platelet-to-lymphocyte ratio (PLR) were also calculated. All measurements were completed in a blinded fashion regarding diagnosis and using standard laboratory methods.

### Statistical Analysis

Continuous variables are expressed as mean ± standard deviation and were compared by variance analysis. Within-group comparisons were determined using one-way repeated-measures analysis of variance (ANOVA) with Tukey’s *post hoc* test. Categorical variables are expressed as frequency (percentage) and were compared using a chi-square test. The risk of AD or MCI was assessed using binary logistic regression analysis. The receiver operating characteristic (ROC) curves of AD- and MCI-related routine blood parameters were established, and the area under the curve (AUC) was calculated to evaluate the diagnostic efficiency of each parameter. The Youden index was calculated, and the corresponding point of the maximum Youden index was considered the best cutoff value. All statistical analyses in this study were performed with SPSS 22.0 software (IBM SPSS Inc., Chicago, IL, United States), and statistical significance was set at *p* < 0.05.

## Results

### Baseline Characteristics

**FIGURE 1:**
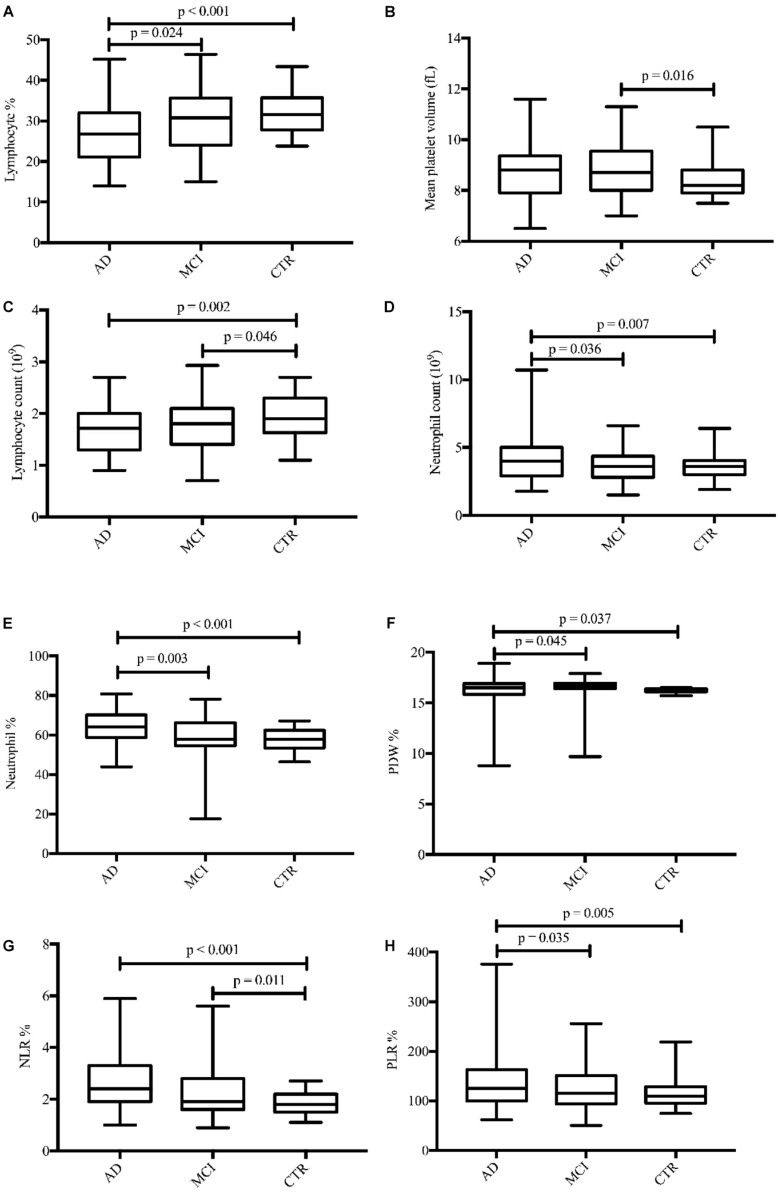
Boxplots for the eight parameters **(A–H)** with significant differences in concentrations between diagnostic groups. AD, Alzheimer’s disease; MCI, mild cognitive impairment; CTRs, controls; PDW, platelet distribution width; NLR, neutrophil-to-lymphocyte ratio; PLR, platelet-to-lymphocyte ratio.

In total, 56 AD patients (23 men; mean age, 69.04 ± 9.05 years), 57 MCI patients (30 men; mean age, 70.67 ± 9.26 years), and 59 healthy controls (24 men; mean age, 68.12 ± 5.81 years) were included in our study. There were no significant differences in age and sex among the groups (Table 1). Seventeen routine blood parameters were included in our study: 14 were recorded directly from laboratory reports and 3 (NLR, LMR, and PLR) were calculated from the report results. There were eight parameters with significant differences between the groups. When the groups were compared, the lymphocyte% tended to gradually increase and the lymphocyte% level was significantly lower in AD patients than in MCI patients. The MPV value of MCI patients was higher than that of controls, but there was no significant difference between the other groups. The lymphocyte count pattern was similar to that of the lymphocyte%, with the lymphocyte count significantly lower in AD and MCI patients than in controls. The neutrophil count was highest in AD patients, and there was a significant difference versus the other two groups (Figures 1A–D). The neutrophil% change showed the same result as the neutrophil count, with the neutrophil% level significantly higher in AD patients versus the other two groups. The PDW value was higher in MCI patients than in AD patients and controls, and there was a significant difference between AD patients and controls. The changes in the NLR and PLR were similar among the three groups, although AD patients showed higher levels than MCI patients, and the difference was significant versus controls (Figures 1E–H).

**TABLE 1:** Blood routine analytes associated with clinical state in the discovery phase.

**Analytes**	**AD (*n* = 56)**	**MCI (*n* = 57)**	**CTR (*n* = 59)**	**AD vs. CTR**	**AD vs. MCI**	**MCI vs. CTR**
	
	**Mean ± SD**	**Mean ± SD**	**Mean ± SD**	***P* value**	***P* value**	***P* value**
Age (years, mean ± SD)	69.04 (9.05)	70.67 (9.26)	68.12 (5.81)	0.517	0.346	0.078
Man, *n*(%)	23 (50.0)	30 (52.63)	24 (40.68)	0.966	0.166	0.185
WBC count(10^9^/L)	6.53 (2.14)	7.21 (9.25)	6.07 (1.16)	0.155	0.593	0.351
Neutrophil%	64.01 (7.97)	58.85 (10.04)	57.87 (5.39)	< 0.001^∗^	0.003^∗^	0.511
Lymphocyte%	27.10 (7.34)	30.32 (7.60)	31.97 (4.90)	< 0.001^∗^	0.024^∗^	0.166
Monocyte%	6.76 (2.13)	7.36 (2.76)	7.39 (1.61)	0.076	0.197	0.952
Neutrophil count(10^9^/L)	4.28 (1.82)	3.66 (1.18)	3.57 (0.81)	0.007^∗^	0.036^∗^	0.599
Lymphocyte count(10^9^/L)	1.69 (0.44)	1.78 (0.47)	1.94 (0.38)	0.002^∗^	0.295	0.046^∗^
Monocyte count(10^9^/L)	0.43 (0.18)	0.44 (0.19)	0.45 (0.12)	0.510	0.797	0.746
Erythrocytes count (10^12^/L)	4.47 (0.44)	4.36 (0.44)	4.40 (0.38)	0.344	0.194	0.642
MCV (fl)	92.18 (4.17)	91.35 (9.90)	93.55 (4.04)	0.076	0.568	0.119
MCH (fl)	30.88 (1.47)	30.89 (1.85)	31.28 (1.30)	0.125	0.973	0.192
RDW (fl)	13.37 (1.30)	13.19 (0.72)	13.10 (0.70)	0.164	0.179	0.489
Platelet count(10^12^/L)	217.64 (57.48)	203.21 (53.93)	218.78 (37.14)	0.899	0.171	0.072
MPV (fl)	8.68 (1.09)	8.78 (1.0)	8.40 (0.63)	0.099	0.585	0.016^∗^
PDW (fl)	15.49 (2.69)	16.33 (1.55)	16.23 (0.22)	0.037^∗^	0.045^∗^	0.639
LMR	4.32 (1.67)	4.33 (1.63)	4.52 (1.24)	0.473	0.974	0.330
NLR	2.61 (1.04)	2.25 (1.01)	1.88 (0.44)	< 0.001^∗^	0.061	0.011^∗^
PLR	141.83 (62.38)	120.84 (39.80)	116.31 (28.65)	0.005^∗^	0.035^∗^	0.482

*WBC, white blood cell; RBC, red blood cell; MCV, mean corpuscular volume; MCH, mean corpuscular hemoglobin; RDW, red cell distribution width; MPV, mean platelet volume; PDW, platelet distribution width; LMR, lymphocyte-to-monocyte ratio; NLR, neutrophil-to-lymphocyte ratio; PLR, platelet-to-lymphocyte ratio.*

### Biomarkers Differentially Expressed Between Groups

#### AD Patients Versus Healthy Controls

**FIGURE 2:**
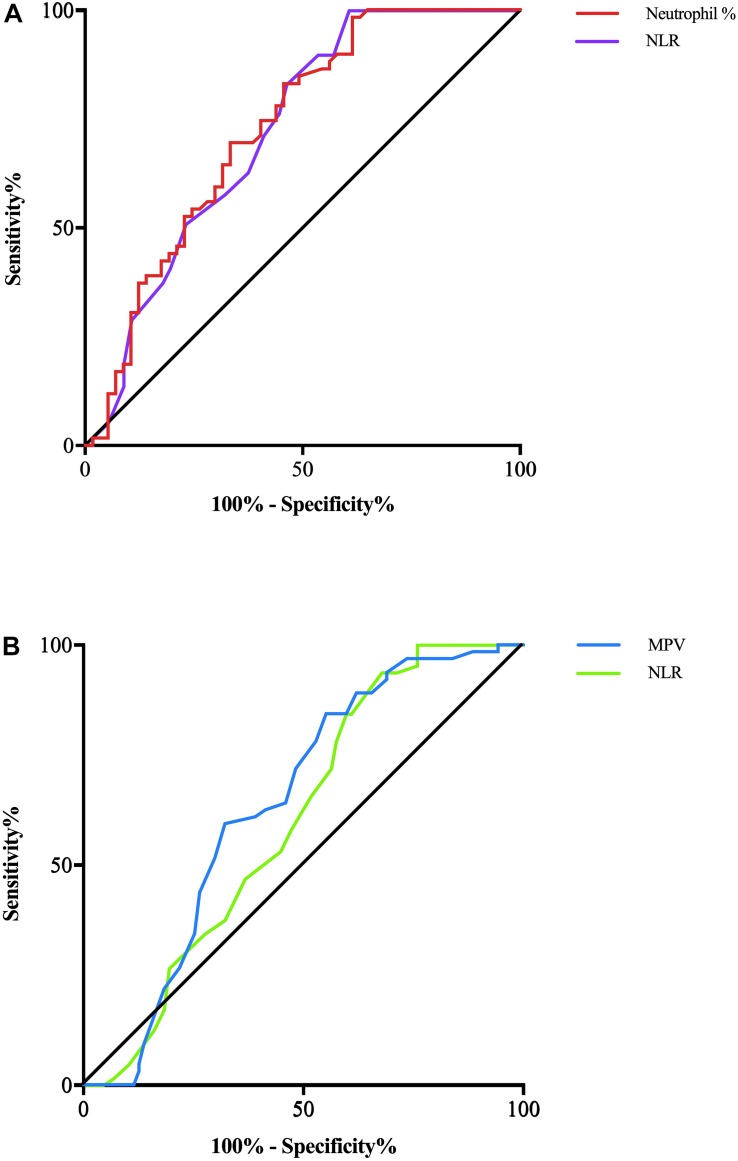
**(A)** AD patients and CTRs are differentiated by the NLR with a predictive power (AUC) of 0.721 (violet line) and by the neutrophil% with a predictive power (AUC) of 0.728 (red line). **(B)** MCI patients and CTRs are differentiated by the NLR with a predictive power (AUC) of 0.601 (green line) and by the MPV with a predictive power (AUC) of 0.690 (blue line).

In terms of parameters, the following were significantly different between AD patients and healthy controls: neutrophil% (64.01 ± 7.97 vs. 57.87 ± 5.39, *p* < 0.001), lymphocyte% (27.10 ± 7.34 vs. 31.97 ± 4.90, *p* < 0.001), neutrophil count (4.28 ± 1.82 vs. 3.57 ± 0.81 × 10^9^/L, *p* = 0.007), lymphocyte count (1.69 ± 0.44 vs. 1.94 ± 0.38 × 10^9^/L, *p* = 0.002), PDW (15.49 ± 2.69 vs. 16.23 ± 0.22, *p* = 0.037), NLR (2.61 ± 1.04 vs. 1.88 ± 0.44, *p* < 0.001), and PLR (141.83 ± 62.38 vs. 116.31 ± 28.65, *p* = 0.005). Binary logistic regression showed that neutrophil% [odds ratio (OR) 1.34, 95% confidence interval (CI) 1.03–1.75, *p* = 0.031] and NLR (OR 6.27, 95% CI 3.98–9.82, *p* = 0.003) were independently associated with AD (Table 2). The diagnostic values for distinguishing healthy controls from AD were as follows: for the NLR, specificity of 53.57% and sensitivity of 83.05% (cutoff value 2.35, AUC 0.721, 95% CI 0.627–0.816, *p* < 0.001), and for the neutrophil%, specificity of 66.67% and sensitivity of 69.49% (cutoff value 60.9%, AUC 0.728, 95% CI 0.636–0.821, *p* < 0.001) (Figure 2A).

**TABLE 2:** Multivariate models for distinguishing between diagnostic groups.

**Predictor**	**AD vs. CTR**		**AD vs. MCI**		**MCI vs. CTR**	
			
	**OR (95% CI)**	***P* value**	**OR (95% CI)**	***P* value**	**OR (95% CI)**	***P* value**
Neutrophil%	1.34 (1.03–1.75)	0.031	n/a	n/a	n/a	n/a
NLR	6.27 (3.98–9.82)	0.003	n/a	n/a	1.93 (1.07–3.47)	0.028
PDW	n/a	n/a	1.22 (1.01–1.47)	0.042	n/a	n/a
MPV	n/a	n/a	n/a	n/a	1.67 (1.04–2.70)	0.036

*MPV, mean platelet volume; PDW, platelet distribution width; NLR, neutrophil-to-lymphocyte ratio.*

#### MCI Patients Versus Healthy Controls

The following parameters were significantly different between MCI patients and healthy controls: lymphocyte count (1.78 ± 0.47 vs. 1.94 ± 0.38 × 10^9^/L, *p* = 0.046), MPV (8.78 ± 1.0 vs. 8.40 ± 0.63, *p* = 0.016), and NLR (2.25 ± 1.01 vs. 1.88 ± 0.44, *p* = 0.011). Binary logistic regression showed that NLR (OR 1.93, 95% CI 1.07–3.47, *p* = 0.028) and MPV (OR 1.67, 95% CI 1.04–2.70, *p* = 0.036) were independently associated with MCI (Table 2). In terms of diagnostic value, the MPV distinguished healthy controls from MCI individuals with a specificity of 51.72% and a sensitivity of 71.88% (cutoff value 8.75, AUC 0.638, 95% CI 0.550–0.726, *p* = 0.004), whereas NLR had a specificity of 40.24% and sensitivity of 84.38% (cutoff value 2.34, AUC 0.601, 95% CI 0.512–0.690, *p* < 0.03) (Figure 2B).

#### AD Patients Versus MCI Patients

The expression levels of other biomarker differed between AD and MCI patients. Neutrophil% (64.01 ± 7.97 vs. 58.85 ± 10.04, *p* = 0.003), lymphocyte% (27.10 ± 7.34 vs. 30.32 ± 7.60, *p* = 0.024), neutrophil count (4.28 ± 1.82 vs. 3.66 ± 1.18 × 10^9^/L, *p* = 0.036), PDW (15.49 ± 2.69 vs. 16.33 ± 1.55, *p* = 0.045), and PLR (141.83 ± 62.38 vs. 120.84 ± 39.80, *p* = 0.035) were significantly different between patients with AD and MCI. Logistic regression showed that PDW (OR 1.22, 95% CI 1.01–1.47, *p* = 0.042) was independently associated with AD (Table 2). However, we did not obtain effective diagnostic biomarkers to distinguish AD from MCI.

## Discussion

Alzheimer’s disease is a neurodegenerative disease characterized by an obscure onset, progressive memory disorders, cognitive dysfunction, and mental behavioral abnormalities. Its diagnosis mainly relies on clinical symptoms and neuropsychological assessment due to the lack of reliable objective indicators. Before the symptom severity reaches the clinical diagnostic criteria of AD, the disease may have been ongoing for decades.

There have been many studies of CSF and plasma biomarkers in AD patients. Among the biomarkers analyzed, the Aβ_1__–__42_:Aβ_1__–__40_ ratio has shown potential as a screening or diagnostic biomarker in several studies (Petersen and O’Bryant, 2019; Vergallo et al., 2019). Furthermore, the plasma Aβ_1__–__42_:Aβ_1__–__40_ ratio is reduced in AD patients and has good diagnostic accuracy (Ovod et al., 2017). Plasma tau protein levels are reported to be elevated in patients with AD compared with controls (O’Bryant et al., 2011), and Mielke et al. (2017) suggested that plasma tau could be a non-disease-specific screening marker. Plasma neurofilament light chain (NfL) levels significantly correlate with CSF levels (Kuhle et al., 2016), with Weston et al. (2017) finding a marked elevation in plasma NfL in AD patients, which could be comparable to the plasma Aβ_1__–__42_:Aβ_1__–__40_ ratio in terms of diagnostic efficiency. Stevenson et al. (2017) suggested that erythrocytes could reflect the intrinsic pathophysiological characteristics of dementia syndromes, including AD. They also pointed out that erythrocyte morphology and erythrocyte protein levels, such as Hsp90, calpain-1, and IgG A, were promising preclinical blood biomarkers for AD. Heneka et al. (2015) examined 53 types of inflammatory proteins in the plasma of 401 patients with cognitive impairment and compared them with 259 healthy controls. The results showed that FB (factor B) and FH (factor H) could predict the progression of MCI to AD. Baird et al. (2015) systematically reviewed the serum and CSF biomarkers with early diagnostic value for AD. Although the application of these biomarkers shows considerable promise, they cannot be widely used in patients and have poor repeatability due to various limitations, particularly the sampling method, which is traumatic for CSF. A methodology involving the detection of some protein components in plasma cannot be applied to patients in developing countries because of the experimental method or high cost. Therefore, biomarkers for the diagnosis of AD are still in the exploratory stage.

Considering the role of inflammation in the pathogenesis of AD, we hypothesized that routine blood parameters in AD patients could have diagnostic and predictive value and 17 routine blood biomarkers were obtained from 56 AD patients, 57 MCI patients, and 59 healthy controls. Eight parameters showed significant differences between the groups, and five were independently associated with AD and MCI. In this study, some inflammatory biomarkers had clear changes among the three groups. Lymphocyte ratio and count values were significantly decreased in AD patients, followed by MCI patients, and there were significant differences compared with the controls, which suggested that multiple pathological mechanisms may influence lymphocyte proliferation, including inflammation and oxidative stress reactions (Pluta et al., 2018). Among the factors playing a role in the inflammatory mechanism of AD, lymphocytes migrate to the brain through the blood–brain barrier. In addition, Pluta and Ulamek-Koziol (2019) reviewed previous studies and suggested that blood components such as lymphocytes, platelets, and erythrocytes can be easily used as biomarkers for pre-clinical and definite clinical diagnosis of AD. Shad et al. (2013) reached the same conclusion and believed that the peripheral blood lymphocyte levels of AD patients were constantly low. In contrast to lymphocytes, the neutrophil ratio and count were increased in AD and MCI patients, which may also suggest that the increased neutrophils were associated with inflammation occurrence and progression (Scali et al., 2002). Previous studies confirmed that the NLR and PLR were elevated in AD and other neurodegenerative diseases (Rembach et al., 2014; Tak and Sengul, 2019), which was consistent with our finding that the NLR and PLR were significantly higher in AD and MCI patients. MPV is a determining factor of platelet function, and an elevated MPV is correlated with vascular inflammation. Koc et al. (2014) reported that MPV levels were higher in AD patients. Yesil et al. (2012) also considered that an increased MPV was associated with vascular risk in AD. Our results also showed an elevated MPV in AD and MCI patients, although the MPV elevation was only significant in MCI patients compared with controls. PDW is another platelet indicator that reflects variations in platelet size. Wang et al. (2013) found that a decreased PDW was associated with MCI and AD. Liang et al. (2014) also reported that the PDW was decreased in both vascular dementia and AD, and our results also suggested that the PDW was significantly decreased in AD patients. Furthermore, after the use of ROC curve analysis to identify the biomarkers with the highest diagnostic value, NLR (AUC 0.721) and neutrophil% (AUC 0.728) distinguished AD patients and healthy controls, whereas NLR (AUC 0.601) and MPV (AUC 0.638) distinguished MCI individuals and healthy controls.

An increased neutrophil count is often associated with inflammation occurrence, progression, and severity, whereas a decreased lymphocyte count, as part of the immune regulatory barrier, is associated with the body’s stress response. Therefore, the NLR, as a combined inflammatory biomarker, integrates information from the two leukocyte subtypes. In particular, it avoids the disadvantage of an absolute value of a single leukocyte subtype, which may be affected by infection or dehydration, and has higher clinical significance than other independent inflammatory biomarkers (Petrone et al., 2019). In recent years, more and more studies have confirmed that the NLR is associated with atherosclerosis and stroke prognosis (Kocaturk et al., 2019). As for the association of the NLR with neurodegenerative diseases, Akil et al. (2015), by comparing NLR levels between 51 Parkinson’s disease patients and 50 healthy controls, suggested that the NLR was significantly higher in Parkinson’s disease patients. Nakamura et al. (2010), who studied the role of systemic inflammation in the pathogenesis of amyotrophic lateral sclerosis (ALS), found that the NLR was higher in patients with ALS and concluded that it could be a biomarker to predict negative prognosis in ALS. Ohtani et al. (2018) found that the NLR was higher in MCI patients than in healthy controls. The NLR can also act as a predictor of cognitive dysfunction in carotid endarterectomy patients (Halazun et al., 2014). Rembach et al. (2014) determined that the NLR had a limited association with cognitive decline and that it may reflect AD-related inflammatory processes in the periphery. All of these conclusions were consistent with our findings that the NLR was higher in both AD and MCI patients. Kalelioglu et al. (2017) speculated that a higher NLR may be a consequence of more active or more recent inflammatory disease processes in MCI (relative to AD). Our results also agree with those of Kuyumcu et al. (2012), who suggested that elderly people with AD have a higher NLR than healthy controls and that the optimum NLR cutoff for AD was 2.48. To sum up, the NLR could be a very promising auxiliary diagnostic biomarker for AD and MCI.

Mean platelet volume is another inflammatory marker in various diseases (Korniluk et al., 2019). Increased MPV in patients with AD may point to platelet dysfunction because MPV is an indicator of increased *in vivo* platelet activation. Hence, platelets could be the link between vascular risk factors and AD. Yesil et al. (2012) also reported that an increased MPV reflected vascular risk in AD. Chen et al. (2017), comparing the routine blood parameters of 92 patients with AD and 84 healthy controls, determined that MPV levels were significantly elevated in AD patients. Koc et al. (2014) found that MPV levels were significantly elevated in AD patients and were associated with cognitive impairment severity. In our study, we also confirmed that the MPV was higher in MCI patients and had an optimal diagnostic value in distinguishing MCI patients from healthy controls.

Neutrophils play an important role in the non-specific immune system of the body. The neutrophil% reflects the neutrophil level and is less affected by the body’s blood volume; in addition, its stability is high, which has a certain clinical value (Shaul and Fridlender, 2017). Neutrophil elevation in peripheral blood of patients with AD has been reported, and Vida et al. (2017) believe that oxidative stress and damage parameters, as well as peripheral cytokine release, are associated with elevated neutrophils in AD patients. It was also found that extravasated neutrophils were present in areas with Aβ deposits and that they contributed to AD pathogenesis and cognitive impairment in a mouse model (Zenaro et al., 2015). As a result of chronic systemic inflammation, the percentage of neutrophils in peripheral blood of AD patients increases, and this tendency increases with age. Dong et al. (2018) suggest that the neutrophil phenotype may be related to the rate of cognitive decline and may thus constitute an innovative and prognostic blood biomarker in AD patients. In our study, we also found that the neutrophil% was significantly higher in the AD and MCI groups, and we confirmed that the neutrophil% has diagnostic value in distinguishing AD patients and healthy controls.

The limitations of the present study include a relatively small sample size for each group and the cross-sectional design. The use of a single peripheral blood sample may have increased the risk of random errors. In addition, the identification of inflammation with other inflammatory biomarkers will add valuable information and support our findings.

## Conclusion

Our study improves our understanding of the role played by inflammation in cognitive impairment. Biomarkers in routine blood samples may correlate with cognitive impairment, with the NLR, neutrophil%, and MPV potentially useful for the identification of patients with AD and MCI. The diagnostic efficacy of these biomarkers should be validated in a larger population in future work.

## Data Availability Statement

All datasets generated for this study are included in the article/supplementary material.

## Ethics Statement

All procedures performed in the studies involving human participants were in accordance with the ethical standards of the institutional and/or national research committee and with the 1964 Helsinki Declaration and its later amendments or comparable ethical standards. All participants were informed of the purpose of the study.

## Author Contributions

XD contributed to the study design, analysis, and interpretation of data, and drafting of the manuscript. JS contributed to acquisition of data. DZ and JN contributed to critical revision of the manuscript for important intellectual content.

## Conflict of Interest

The authors declare that the research was conducted in the absence of any commercial or financial relationships that could be construed as a potential conflict of interest.
